# Tea and Risk of Age-Related Cataracts: A Cross-Sectional Study in Zhejiang Province, China

**DOI:** 10.2188/jea.JE20150223

**Published:** 2016-11-05

**Authors:** Yan Sheng, Fan He, Jun-Fen Lin, Wei Shen, Yin-Wei Qiu

**Affiliations:** 1 The First Affiliated Hospital, College of Medicine, Zhejiang University, Zhejiang Province, People’s Republic of China; 2 Zhejiang Provincial Center for Disease Control and Prevention, Zhejiang Province, People’s Republic of China

**Keywords:** tea, cataract, cross-sectional study, odds ratio

## Abstract

**Background:**

The antioxidant properties of tea extracts are considered to be effective in protecting against cataracts. However, there is still insufficient epidemiological knowledge about the protective effects of different types of tea on age-related cataracts.

**Methods:**

The data was derived from the Zhejiang Major Public Health Surveillance (ZJMPHS) Program on health and related factors in the elderly. The relationships between consumption of different types of tea and risk of age-related cataracts were assessed after adjusting for related covariates.

**Results:**

The prevalence of age-related cataracts in this study population was 4.4% (409/9343). After adjustment for potential confounders, tea drinking was associated with reduced risk of age-related cataracts (adjusted odds ratio [OR] 0.65; 95% confidence interval [CI], 0.47–0.91). Compared to nondrinkers, green tea drinkers had a significantly reduced risk of cataracts (adjusted OR 0.58; 95% CI, 0.40–0.85). Average tea consumption of 14–27 cups (adjusted OR 0.55; 95% CI, 0.33–0.93) and over 28 cups (adjusted OR 0.58; 95% CI, 0.34–0.99) per week had a protective effect against cataracts in comparison to no consumption. In addition, ingesting a moderate concentration of tea significantly decreased the risk of cataract compared to no consumption (adjusted OR 0.43; 95% CI, 0.27–0.71).

**Conclusions:**

Tea ingestion was associated with reduced risk of age-related cataracts. In light of these findings, we suggest that reasonable tea consumption (ie, favoring green tea and consuming an average of over 500 mL per day at moderate concentration) should offer protection against age-related cataracts.

## INTRODUCTION

Cataracts are the main cause of blindness and are responsible for approximately 50% of the number of people in China who are blind, or around 2.5 million people, and the incidence is increasing.^1^ Age-related cataracts are the predominant type, and over 80% of cataract-disabled people in China are aged 65 years or older.^2^ Age-related cataracts are considered a multifactorial ophthalmic disease that might be related to the environment, nutrition, metabolism, genetics, and other unknown factors. Many factors, such as smoking, alcohol use, ultraviolet radiation exposure, diabetes, diarrhea, and some nutritional factors significantly increase the risk of age-related cataracts.^3^ Other factors, including consumption of many types of vegetables, fruits, and micronutrients, might reduce the risk of this disease.^4^ Green and black teas are considered to be protective against cataracts, with a proposed mechanism related to the antioxidant properties of compounds found in tea extracts, such as catechin (C), catechin gallate (CG), epicatechin gallate (ECG), epigallocatechin gallate (EGCG), theaflavins (TFs), and thearubigins (TGs).^5^ To our knowledge, however, only a few epidemiologic research studies have examined the relationship between tea consumption and risk of cataracts.^6^^,^^7^ Further, epidemiological knowledge about the protective effects of different types of tea on age-related cataracts remains insufficient. The relationship between consumption and concentration of tea and risk of cataracts is also not well understood. The lack of such information hinders the establishment of guidelines for tea intake to prevent or delay the development of cataracts.

To address this knowledge gap, we conducted a cross-sectional study to identify the effect of tea drinking on the risk of age-related cataracts by adjusting for demographics, occupational exposure, underlying diseases, lifestyle, micronutrient intake, and other risk-related factors.

## METHODS

### Study population

Participants in the Zhejiang Major Public Health Surveillance (ZJMPHS) Program on health and related factors in the elderly who had undergone interviews at a baseline survey in 2014 were included in these analyses. The program was conducted in six counties, which were randomly selected from among 90 counties in Zhejiang Province, an economically developed province in eastern China that produces abundant quantities of tea. For each county, over 1500 permanent residents aged ≥60 years were investigated using a two-stage sampling strategy. From October to December in 2014, a self-designed questionnaire was used to interview participants face to face. The questionnaire included information concerning tea ingestion (including types, concentration, and typical consumption), demographics, occupational exposure, underlying diseases (before the onset of cataract), lifestyle (such as alcohol consumption, cigarette smoking, and physical activity), and micronutrient intake.

We defined patients as having an age-related cataract when they had lens opacity with a best-corrected visual acuity of less than 20/30 in either eye and when all other causes of cataracts, such as trauma, steroids, intraocular inflammation, or intraocular surgery, had been excluded. The cause of lens opacity could be due to nuclear, cortical, posterior subcapsular, or mixed-type cataracts. Those participants who underwent cataract extraction in either eye without experiencing other ocular diseases were also defined as having age-related cataracts.

Each participant was given a preliminary screening of the patient’s visual acuity (wearing spectacles if necessary) and through the use of a slit-lamp examination during an annual routine medical examination. Those participants with a visual acuity of less than 20/30 and with varying degrees of lens opacity in either eye were examined further at a local hospital. The participants underwent a comprehensive ocular examination, which included best-corrected visual acuity testing with refraction, a slit-lamp examination of the lens and anterior segment, a fundus examination by direct ophthalmoscopy or by a +78D/+90D non-contact lens, and by measuring intraocular pressure with a non-contact tonometer. If an alternate senile ocular disease that might cause vision impairment, such as age-related macular degeneration, was detected, the lens opacity needed to be assessed for severity sufficient to reduce visual acuity to 20/30 or worse when it was considered alone. All examinations were carried out by experienced ophthalmologists.

A total of 9353 elderly patients aged ≥60 years responded to the investigation. There was at least some information for these patients related to the diagnosis of cataract and tea ingestion. Ten patients with cataracts that were considered to be congenital or secondary to ocular trauma were excluded. Ultimately, a total of 9343 participants were included in the analysis.

Written informed consent was obtained from all participants, and the program was approved by the Ethics Committee of Zhejiang Provincial Centre for Disease Control and Prevention.

### Assessment of tea intake

In the questionnaire, the participants were asked whether they drink tea in their daily life. For those who drank tea, the main types, intake, and concentration of tea were further investigated. Tea was classified into green, black, and others (including oolong, pu’er, scented, and fruit teas). Regarding tea intake, participants were asked about average cups per week (one cup equal to 250 mL) and were then categorized into four groups: none, <14 cups, 14 to 27 cups, and ≥28 cups. Concentration was classified as zero, light (1 teaspoon, or about 1–2 grams of tea per cup of water), moderate (1.5 teaspoons, or about 3–4 grams of tea per cup of water) and thick (2 teaspoons, or about 5 grams of tea per cup of water).

### Other covariates

Demographic information, including sex, ethnic group, age, body mass index (BMI), marital status (unmarried, married, widowed, or divorced), education level (illiterate or semiliterate, primary school, junior high school, or high school graduation or higher), health insurance, and household income, was collected via questionnaires. Occupational exposures that may be harmful to the eyes and increase the chance of development of cataracts, such as exposure to pesticides, dust, irritating gases, and heavy metals, and family history of cataract were reviewed. Presence of underlying diseases, including high blood pressure, hyperlipidemia, and diabetes, was identified by checking medical records. Lifestyle information, including smoking status (nonsmokers, current smokers, or ex-smokers) and alcohol drinking status (nondrinkers, current drinkers, or ex-drinkers), was investigated. Moreover, physical activity (none, 1–3 periods of exercise per week, or >3 times per week) was also taken into account, since it might increase the risk of cataracts as a result of prolonged exposure to ultraviolet light.^8^ Subjects were also interviewed to collect information on their regular peroral intake of micronutrient supplements, such as multivitamins, vitamin A, β-carotene, vitamin B, vitamin C, vitamin E, calcium, and iron. Moreover, the form (liquid, capsule, or tablet) and brand of any micronutrient supplements taken, as well as the frequency of intake, were recorded.

### Statistical analysis

Data were reviewed for outliers using descriptive analyses. Distributions of tea ingestion and other covariates among participants with and without cataracts were compared using a chi-square test. A multivariate logistic regression was used to calculate adjusted odds ratios (ORs) and 95% confidence intervals (CIs) as measures of association between tea ingestion and cataracts. Stratified analyses and interaction terms were used to examine effect modification of tea consumption by family history of cataracts. All analyses were performed using PASW software, version 18.0.0 (SPSS Inc., Chicago, IL, USA).

## RESULTS

Of the 9343 study participants, 409 (4.4%) were diagnosed with age-related cataracts. Characteristics of participants with and without cataracts are shown in Table 1. The proportion of participants with cataracts was higher for the female, ethnic Han, widowed, and elderly categories. Those with cataracts were more likely to have higher BMI; to have dust, irritant gas, and heavy metal exposure; to engage in physical activity; and to use β-carotene supplements. Moreover, they seemed to have lower income, be nonsmokers, nondrinkers of alcohol, and consumed less tea (especially green tea).

**Table 1.** Distribution of patients with and without cataracts according to selected covariates

Characteristic	Cataract (*n* = 409)		No cataracts (*n* = 8934)		*P* value
	
	*n*	%	*n*	%	
Male	141	34.5	4383	49.1	<0.001
Ethnic Han	402	98.3	8571	96.0	0.018
Age, years					<0.001
<65	63	15.4	2937	32.9	
65–69	93	22.7	2235	25.0	
70–74	82	20.0	1377	15.4	
75–79	84	20.5	1201	13.4	
80–84	58	14.2	797	8.9	
≥85	29	7.1	387	4.3	
BMI, kg/m^2^					0.021
18.5–24.99	233	59.4	5513	66.2	
<18.50	31	7.9	515	6.2	
>24.99	128	32.7	2301	27.6	
Education					0.260
Illiterate or semiliterate	188	46.0	4548	50.9	
Primary school	189	46.2	3744	41.9	
Junior high school	26	6.4	537	6.0	
High school graduation or higher	6	1.5	103	1.2	
Marital status					<0.001
Unmarried	3	0.7	150	1.7	
Married	267	65.3	6746	75.7	
Widowed	136	33.3	1979	22.2	
Divorced	3	0.7	39	0.4	
Pesticide exposure	235	57.7	5457	61.1	0.170
Dust exposure	37	9.3	209	2.5	<0.001
Irritant gas exposure	4	1.0	21	0.3	0.006
Heavy metal exposure	9	2.3	18	0.2	<0.001
Family income, 1000 ¥ per year					<0.001
<10	82	20.1	1972	22.1	
10–49	255	62.5	4346	48.8	
50–99	46	11.3	1290	14.5	
100–149	16	3.9	807	9.1	
150–199	3	0.7	338	3.8	
≥200	6	1.5	161	1.8	
Health insurance	407	99.5	8897	99.6	0.789
High blood pressure	155	37.9	3968	44.4	0.009
Hyperlipidemia	27	6.6	426	4.8	0.092
Diabetes	40	9.8	737	8.2	0.273
Family history of cataracts	17	4.8	24	0.3	<0.001
Smoking					<0.001
Current smoker	48	11.7	1949	21.8	
Ex-smoker	40	9.8	883	9.9	
Nonsmoker	321	78.5	6102	68.3	
Alcohol drinking					0.024
Current drinker	81	19.8	2273	25.4	
Ex-drinker	35	8.6	823	9.2	
Nondrinker	293	71.6	5838	65.3	
Physical activity					<0.001
None	267	65.3	7331	82.1	
1–3 times per week	31	7.6	289	3.2	
>3 times per week	111	27.1	1314	14.7	
Vitamin A^a^	2	0.5	16	0.2	0.165
β-carotene^a^	2	0.5	3	0.03	<0.001
Vitamin B^a^	1	0.2	8	0.1	0.326
Vitamin C^a^	2	0.5	11	0.1	0.053
Vitamin E^a^	1	0.2	15	0.2	0.719
Calcium tablets^a^	42	10.3	731	8.2	0.132
Iron^a^	1	0.2	14	0.2	0.669
Tea consumption	58	14.2	2469	27.6	<0.001
Type					<0.001
Nondrinker	351	85.8	6465	72.4	
Green tea	43	10.5	1909	21.4	
Black tea	8	2.0	480	5.4	
Other	7	1.7	80	0.9	
Tea intake, cups per week					<0.001
None	351	85.8	6465	72.4	
<14	19	4.6	532	6.0	
14–27	21	5.1	971	10.9	
≥28	18	4.4	966	10.8	
Concentration of tea					<0.001
Zero	351	85.8	6465	72.4	
Light	20	4.9	582	6.5	
Moderate	24	5.9	1457	16.3	
Thick	14	3.4	430	4.8	

BMI, body mass index.

^a^Derived from only dietary supplements and not from diet.

About 14% of participants with cataracts and 28% of those without cataracts reported drinking tea, with a significant association between tea consumption and cataracts (OR 0.65; 95% CI, 0.47–0.91) after adjustment for sex; ethnic group; age; BMI; education; marriage status; exposure to pesticides, dust, irritant gas, and heavy metals; family income; health insurance status; presence of high blood pressure, hyperlipidemia, or diabetes; family history of cataract; smoking, alcohol drinking, and physical activity status; and intake of vitamin A, β-carotene, vitamin B, vitamin C, vitamin E, calcium tablets, and iron. Participants who were female, had dementia, were overweight, and had a higher education level had a significantly higher risk of cataracts. Pesticide (OR 1.34; 95% CI, 1.04–1.72), dust (OR 2.10; 95% CI, 1.29–3.40), and heavy metal (OR 11.83; 95% CI, 4.55–30.75) exposure during the work period and having a family history of cataracts (OR 14.80; 95% CI, 6.98–31.37) were associated with increased risk of cataracts after adjustment for related covariates. Compared to no physical activity, exercise 1–3 times per week (OR 3.00; 95% CI, 1.92–4.67) and over 3 times per week (OR 1.73; 95% CI, 1.30–2.29) also increased the risk of cataracts (Table 2). Stratified analyses showed a family history of cataracts and tea consumption had an independent effect on cataracts, with odds ratios of 13.12 (95% CI, 6.50–26.46) and 0.45 (95% CI, 0.33–0.60), respectively. The estimated odds ratio for the combination of family history and tea consumption was 8.48 (95% CI, 1.64–43.87), which was less than that expected from adding (OR 12.57) and more than that from multiplying (OR 5.90) their independent associations, suggesting an interaction between family history and tea consumption under the additive and multiplicative risk models.

**Table 2.** Unadjusted and adjusted odds ratios for the associations between selected covariates and cataracts

Parameter	Unadjusted			Multi-adjusted^a^		
	
	OR	95% CI	*P* value	OR	95% CI	*P* value
Male	0.55	0.44–0.67	0.000	0.46	0.32–0.66	0.000
Ethnic Han	2.42	1.14–5.14	0.022	1.62	0.7–3.74	0.261
Age, years						
<65	1.00	Reference		1.00	Reference	
65–69	1.94	1.40–2.68	0.000	2.22	1.53–3.21	0.000
70–74	2.78	1.99–3.88	0.000	3.81	2.57–5.65	0.000
75–79	3.26	2.34–4.55	0.000	5.57	3.71–8.36	0.000
80–84	3.39	2.36–4.89	0.000	4.85	3.02–7.79	0.000
≥85	3.49	2.22–5.49	0.000	5.24	2.94–9.32	0.000
BMI, kg/m^2^						
18.5–24.99	1.00	Reference		1.00	Reference	
<18.50	1.42	0.97–2.09	0.072	1.37	0.90–2.08	0.142
>24.99	1.32	1.06–1.64	0.015	1.34	1.03–1.74	0.031
Education						
Illiterate or semiliterate	1.00	Reference		1.00	Reference	
Primary school	1.22	0.99–1.50	0.058	1.57	1.2–2.05	0.001
Junior high school	1.17	0.77–1.78	0.460	1.97	1.18–3.28	0.009
High school graduation or higher	1.41	0.61–3.25	0.421	2.07	0.74–5.78	0.167
Marital status						
Married	1.00	Reference		1.00	Reference	
Unmarried	0.51	0.16–1.60	0.244	0.59	0.14–2.46	0.470
Widowed	1.74	1.40–2.15	0.000	1.26	0.95–1.66	0.104
Divorced	1.94	0.60–6.33	0.270	4.96	1.42–17.32	0.012
Pesticide exposure	0.87	0.71–1.06	0.170	1.34	1.04–1.72	0.022
Dust exposure	4.01	2.78–5.77	0.000	2.10	1.29–3.40	0.003
Irritant gas exposure	4.05	1.38–11.85	0.011	2.87	0.76–10.84	0.119
Heavy metal exposure	10.74	4.79–24.06	0.000	11.83	4.55–30.75	0.000
Family income, 1000 ¥ per year						
<10	1.00	Reference		1.00	Reference	
10–49	1.41	1.09–1.82	0.008	1.44	1.07–1.95	0.018
50–99	0.86	0.59–1.24	0.413	1.12	0.73–1.72	0.611
100–149	0.48	0.28–0.82	0.007	0.86	0.48–1.57	0.631
150–199	0.21	0.07–0.68	0.009	0.17	0.02–1.23	0.079
≥200	0.90	0.39–2.09	0.799	1.29	0.50–3.38	0.600
Health insurance	0.82	0.20–3.43	0.790	0.75	0.18–3.26	0.705
High blood pressure	0.76	0.62–0.94	0.010	0.51	0.40–0.66	0.000
Hyperlipidemia	1.41	0.94–2.11	0.093	1.36	0.83–2.21	0.225
Diabetes	1.21	0.86–1.69	0.274	1.01	0.66–1.54	0.958
Family history of cataract	17.82	9.48–33.48	0.000	14.80	6.98–31.37	0.000
Smoking						
Nonsmoker	1.00	Reference		1.00	Reference	
Current smoker	0.47	0.34–0.64	0.000	0.82	0.53–1.25	0.346
Ex-smoker	0.86	0.62–1.21	0.383	1.13	0.71–1.8	0.617
Alcohol drinking						
Nondrinker	1.00	Reference		1.00	Reference	
Current drinker	0.71	0.55–0.91	0.007	1.28	0.92–1.77	0.144
Ex-drinker	0.85	0.59–1.21	0.365	1.07	0.68–1.69	0.779
Physical activity						
None	1.00	Reference		1.00	Reference	
1–3 times per week	2.95	1.99–4.35	0.000	3.00	1.92–4.67	0.000
>3 times per week	2.32	1.85–2.92	0.000	1.73	1.30–2.29	0.000
Vitamin A^b^	2.72	0.62–11.88	0.183	3.49	0.67–18.34	0.139
β-carotene^b^	14.54	2.42–87.26	0.003	3.63	0.37–35.14	0.266
Vitamin B^b^	2.72	0.34–21.78	0.346	1.40	0.11–17.4	0.796
Vitamin C^b^	3.96	0.88–17.93	0.074	0.92	0.10–8.67	0.941
Vitamin E^b^	1.45	0.19–10.99	0.720	1.79	0.21–15.17	0.595
Calcium tablets^b^	1.29	0.93–1.79	0.133	1.17	0.79–1.72	0.434
Iron^b^	1.55	0.20–11.83	0.671	2.39	0.30–19.32	0.415
Tea	0.43	0.33–0.57	0.000	0.65	0.47–0.91	0.013

BMI, body mass index; CI, confidence interval; OR, odds ratio.

^a^Adjusted for sex, ethnicity, age, BMI, education, marriage status, pesticide, dust, irritant gas and heavy metal exposure, family income, health insurance, high blood pressure, hyperlipidemia, diabetes, family history of cataract, smoking, alcohol drinking, physical activity, and intake of vitamin A, β-carotene, vitamin B, vitamin C, vitamin E, calcium tablets and iron.

^b^Derived from only dietary supplements and not from diet.

Among the different types of tea, only green tea was associated with a significantly reduced risk of cataracts after adjustment for selected covariates (in contrast to not drinking tea: OR 0.58; 95% CI, 0.40–0.85). Compared to no tea intake, ≥14 and ≥28 cups per week reduced the risk of cataracts, with odds ratios of 0.55 (95% CI, 0.33–0.93) and 0.58 (95% CI, 0.34–0.99), respectively. Moreover, a medium concentration of tea was associated with decreased risk of cataracts, with an adjusted odds ratio of 0.43 (95% CI, 0.27–0.71) (Table 3).

**Table 3.** Unadjusted and adjusted odds ratios for the associations between type, amount, and concentration of tea and cataract

Tea intake	Cataract		No cataracts		Unadjusted			Multi-adjusted^a^		
			
	No.	%	No.	%	OR	95% CI	*P* value	OR	95% CI	*P* value
Type of tea										
None	351	85.8	6465	72.4	1.00	Reference		1.00	Reference	
Green tea	43	10.5	1909	21.4	0.42	0.30–0.57	0.000	0.58	0.40–0.85	0.005
Black tea	8	2.0	480	5.4	0.31	0.15–0.62	0.001	0.80	0.35–1.81	0.591
Other	7	1.7	80	0.9	1.61	0.74–3.52	0.230	1.53	0.58–4.02	0.390
Tea intake, cups per week										
None	351	85.8	6465	72.4	1.00	Reference		1.00	Reference	
<14	19	4.6	532	6.0	0.66	0.41–1.05	0.081	0.92	0.54–1.57	0.765
14–27	21	5.1	971	10.9	0.40	0.26–0.62	0.000	0.55	0.33–0.93	0.025
≥28	18	4.4	966	10.8	0.34	0.21–0.55	0.000	0.58	0.34–0.99	0.044
Concentration of tea										
Zero	351	85.8	6465	72.4	1.00	Reference		1.00	Reference	
Light	20	4.9	582	6.5	0.63	0.40–1.00	0.051	0.90	0.54–1.51	0.698
Moderate	24	5.9	1457	16.3	0.30	0.20–0.46	0.000	0.43	0.27–0.71	0.001
Thick	14	3.4	430	4.8	0.60	0.35–1.03	0.065	1.04	0.56–1.92	0.897

CI, confidence interval; OR, odds ratio.

^a^Adjusted for sex, ethnicity, age, BMI, education, marriage status, pesticide, dust, irritant gas and heavy metal exposure, family income, health insurance, high blood pressure, hyperlipidemia, diabetes, family history of cataract, smoking, alcohol drinking, physical activity, and intake of vitamin A, β-carotene, vitamin B, vitamin C, vitamin E, calcium tablets and iron.

## DISCUSSION

The findings of this study support a significant protective role of tea ingestion against cataracts in older people. Green tea was associated with a reduced risk of cataracts after controlling for potential confounders. Moreover, the results also suggested that a reasonable average daily tea intake >2 cups (500 mL) at a moderate concentration may inhibit age-related cataracts.

Cataracts are a multifactorial ophthalmic disease, and oxidative stress is a proven important initiator of cataract formation. Therefore, a chemical and pharmacological approach, including supplementation with food items and nutrients containing antioxidants,^9^^–^^11^ can delay the onset or retard the progression of cataracts. The antioxidant properties of tea extracts and flavonoids (eg, C, CG, ECG, and EGCG in green tea and TFs and TGs in black tea) are manifested by their ability to inhibit free radical generation, scavenge free radicals, and chelate transition metal ions.^12^ The antioxidant power of these ingredients is believed to be much more efficient than those of vitamin C, vitamin E, and other common antioxidants.^13^^,^^14^ Our study showed that tea ingestion was negatively associated with the risk of age-related cataracts. This epidemiologic evidence is valuable to prevent cataracts in China, since 27% of participants in this study had the habit of drinking tea, which is much more popular than taking other substances containing antioxidants.

The antioxidant activity of tea is mainly due to polyphenols, which are known major constituents of tea.^12^^,^^15^^,^^16^ The protective effects of polyphenols on cataracts have been verified by many in vitro and in vivo studies.^17^^,^^18^ Green, black, and oolong are the three most popular types of tea, which respectively represent the three main manufacturing methods: unfermented, fully fermented, and semifermented.^19^ Although the percentage of polyphenols in black tea (catechins, 3–10%; theaflavins, 3–6%; thearubigens, 12–18%; and flavonols, 6–8%) is less than in green tea (catechins, 30–42%; flavonols, 5–10%; and other flavonoids, 2–4%) due to a different manufacturing procedure which leads to lower antioxidant activity,^20^ black tea is considered to be effective in preventing cataracts as well.^5^^,^^21^ Our univariate and multivariate analyses both supported a role of green tea in decreasing the risk of age-related cataracts. For black tea, despite a protective effect being observed in univariate analysis, the multi-adjusted odds ratio suggested it had no effect on preventing cataracts after accounting for confounding factors. This might be due to an insufficient sample size of participants drinking black tea, which is more often consumed in Western countries.^22^ Therefore, further epidemiological studies on the relationship between black tea consumption and the risk of age-related cataracts are needed.

Many earlier epidemiologic studies identified a relationship between tea consumption and the risk of cardiovascular diseases and cancer in humans.^23^ However, to our knowledge, the investigation by Robertson^7^ was the only epidemiology study to mention an association between tea intake and the risk of cataracts. It reported that consumption of 500 mL of tea per day for a period of 5 years had a protective effect against cataracts (unadjusted OR 0.39). Similarly to the above report, after controlling for confounding factors, we found that more than two cups (250 mL per cup) of tea intake per day on average could significantly decrease the risk of age-related cataracts.

An in vitro study in rats on the concentration of green tea needed for a 50% inhibition of superoxide, hydroxyl, and LP radicals^24^ implied that the protective effect of tea on cataracts might be dependent on concentration. Our study indicated a 57% reduction in risk of cataracts at moderate tea concentration, but no significant association was observed between drinkers with high or low concentration and nondrinkers. This result may be explained by an insufficient sample size of subjects who drank high or low concentrations of tea. The relationship between concentration of tea and cataracts, as well as the mechanism behind it, need to be further studied.

There are at least two strengths of this study. First, the relatively large sample size ensures the stability of the statistical analysis. Second, since cataracts are a disease caused by multiple factors, the effect of tea intake was analyzed by adjusting for various possible contributing factors, including demographics, occupational exposure, underlying diseases, family history, lifestyle, and micronutrient intake, which enhanced the reliability of the findings. In addition, the unified diagnostic and exclusion criteria and standardized questionnaires and measurements also substantially reduced possible bias.

There were also some possible limitations of this study. First, this study had a cross-sectional design; therefore, the causal and temporal associations of tea ingestion and cataracts could not be inferred. If participants with cataracts got into the habit of drinking tea after cataract onset, the strength of the association between tea and cataracts would be underestimated. In other words, the beneficial effect of tea on age-related cataracts might be greater than the estimates presented in this study. In contrast, if the patients ceased drinking tea after cataract onset, the effect of tea ingestion on cataracts would be overestimated. Second, we did not assess the degree of lens opacity or subtype of cataract because different ophthalmologists can draw different conclusions regarding the same patient. However, since lens opacity is clinically relevant to a substantial decline in the patient’s visual function, we focused on cataracts severe enough in degree to cause visual impairment (best corrected visual acuity <20/30) which might affect daily activities.^25^ Finally, we did not do a detailed investigation of the diet of participants, other than taking into account their micronutrient-supplement intake, because of the complex standards for qualifying ingredients of various kinds of foods. For this reason, we could not assess or adjust for the impact of regular food intake on the relationship between tea and cataracts. However, we are in the process of determining these standards and will investigate detailed dietary considerations in future studies.

In conclusion, tea ingestion was associated with reduced risk of age-related cataracts. In light of the findings of this study, we recommend that reasonable methods of tea consumption, such as ingesting green tea at moderate concentration at over 500 mL per day on average, should be encouraged to prevent or retard the progression of age-related cataracts. Moreover, besides age-related cataracts, tea might play a protective role against other types of cataracts. For instance, an earlier study reported that diabetic cataracts could be inhibited by both green and black tea through a hypoglycemic effect, which in turn inhibits the biochemical indicators of pathology.^21^ Therefore, detailed epidemiologic studies of the protective effects of different types of tea against various cataracts and the mechanisms behind this protection should be conducted.
